# Supplementary material for: Comparative Clinical Outcomes of Major Respiratory Viruses in Hospitalized Adults During the Post-Pandemic Period: A Retrospective Cohort Study
Source: Viruses. 2025 Nov 26;17(12):1545. doi: 10.3390/v17121545 (PMC12737539; doi:10.3390/v17121545)
Supplement: Supplementary file 1 [file viruses-17-01545-s001.zip › viruses-4006433-supplementary.pdf]

Supplementary Table S1. Pathogens detected by QIAstat-Dx Respiratory SARS-CoV-2 Panel

**Pathogen**
**SARS-CoV-2**
**Influenza A**
**Influenza A subtype H1N1/2009**
**Influenza A subtype H1**
**Influenza A subtype H3**
**Influenza B**
**Coronavirus 229E**
**Coronavirus HKU1**
**Coronavirus NL63**
**Coronavirus OC43**
**Parainfluenza virus 1**
**Parainfluenza virus 2**
**Parainfluenza virus 3**
**Parainfluenza virus 4**
**Adenovirus**
**Respiratory Syncytial Virus A/B**
**Human Metapneumovirus A/B**
**Rhinovirus/Enterovirus**
Mycoplasma pneumoniae
Chlamydia pneumoniae
Bordetella pertussis

Supplementary Table S2. Laboratory Results at Day 0 and Day 7 (Influenza vs SARS-CoV-2)

IQR, interquartile range; ALC, absolute lymphocyte count; WBC, white blood cell count; CRP, C-reactive

**Laboratory variable**	**Influenza (n=174)**	**SARS-CoV-2 (n=152)**	**p-value**
**WBC (cells/μL), median (IQR) – Day 0**	7420 (4760–11390)	7200 (4335–11335)	0.400*
**WBC (cells/μL), median (IQR) – Day 7**	7270 (4642–11797)	7740 (4275–11072)	0.844*
**ALC (cells/μL), median (IQR) – Day 0**	880 (540–1340)	830 (500–1345)	0.373*
**ALC (cells/μL), median (IQR) – Day 7**	985 (730–1645)	820 (375–1500)	**0.012***
**CRP (mg/L), median (IQR) – Day 0**	63.0 (35.3–142.3)	78.0 (33.0–173.0)	0.396*
**CRP (mg/L), median (IQR) – Day 7**	43.5 (14.8–92.8)	49.0 (17.0–95.0)	0.617*

protein. *Two-sided Mann–Whitney U tests were used to compare groups; p<0.05 was considered statistically significant.
